# Sex Differences in Task Distribution and Task Exposures among Danish House Painters: An Observational Study Combining Questionnaire Data with Biomechanical Measurements

**DOI:** 10.1371/journal.pone.0110899

**Published:** 2014-11-03

**Authors:** Thomas Heilskov-Hansen, Susanne Wulff Svendsen, Jane Frølund Thomsen, Sigurd Mikkelsen, Gert-Åke Hansson

**Affiliations:** 1 Department of Occupational and Environmental Medicine, Bispebjerg University Hospital, Copenhagen, Denmark; 2 Danish Ramazzini Centre, Department of Occupational Medicine, Regional Hospital West Jutland - University Research Clinic, Herning, Denmark; 3 Occupational and Environmental Medicine, Lund University, and University and Regional Laboratories Region Scania, Lund, Sweden; Glasgow University, United Kingdom

## Abstract

**Objectives:**

Sex differences in occupational biomechanical exposures may be part of the explanation why musculoskeletal complaints and disorders tend to be more common among women than among men. We aimed to determine possible sex differences in task distribution and task-specific postures and movements of the upper extremities among Danish house painters, and to establish sex-specific task exposure matrices.

**Methods:**

To obtain task distributions, we sent out a questionnaire to all members of the Painters' Union in Denmark (N = 9364), of whom 53% responded. Respondents reported their task distributions in a typical week. To obtain task exposures, postures and movements were measured in 25 male and 25 female house painters for one whole working day per person. We used goniometers on the wrists, and inclinometers on the forehead and the upper arms. Participants filled in a logbook allowing task-specific exposures to be identified. Percentiles and % time with non-neutral postures were used to characterise postures. Velocity, range of motion, repetitiveness, and variation were used as measures of movement. Cochran-Mantel-Haenszel statistics and unpaired double-sided t-tests with post-hoc Bonferroni correction were used to evaluate sex differences.

**Results:**

Statistically significant (p<0.05) sex differences were revealed in task proportions, but the proportions differed by less than 4%. For task exposures, no statistically significant sex differences were found.

**Conclusions:**

Only minor sex differences were found in task distribution and task exposures regarding postures and movements among Danish house painters. Sex-specific task exposure matrices were established.

## Introduction

Musculoskeletal disorders (MSDs) account for a large part of the population's use of health care, sickness absence, and health related pensioning before the normal retirement age [1]–[3]. Women constitute about half of the working population in many industrialized countries, but female populations are underrepresented in occupational epidemiological studies of MSDs [4]–[6]. It is well known that women report more musculoskeletal complaints in the upper extremities than their male co-workers [7]–[14], and to some extent this may reflect a higher prevalence of work-related MSDs. Three main hypotheses have been proposed to explain this difference between the two sexes. First, women may have a lower threshold for reporting complaints [7], [15]–[19]. Second, occupational biomechanical exposures may be higher among women even within the same job because of sex-segregated tasks [10], [20]–[22], different postures and movements while performing the same task, or higher use of force relative to their maximum [23]. Third, women may be more vulnerable to specific exposures [15], [24]. Increased knowledge on reasons for sex differences could potentially open up perspectives for the prevention of MSDs.

To evaluate the vulnerability hypothesis, studies should focus on MSDs that are as objectively assessed as possible and include men and women who are as equally exposed as possible so that the reporting and exposure hypotheses cannot explain any differences in the occurrence of MSDs. In Denmark, the house painters' trade seems well suited to investigate the vulnerability hypothesis. About 1/3 of the house painters are women, and house painters have exposures that are risk factors for MSDs of the upper extremities [25]. Throughout the period 1998 to 2007, female house painters were 2 to 3 times as likely as male house painters to report claims of MSDs of the upper extremities to the Danish National Board of Industrial Injuries (Rolf Petersen, personal communication). The question is to which degree men and women in the house painters' trade actually have equal biomechanical exposures to the upper extremities.

To evaluate exposure differences between men and women within the same job, task-based exposure assessment may be a practicable approach [26]. Using this approach, the job exposure of an individual is estimated by weighing task exposures (i.e., the specific exposures to a specific body part which result from performing a specific task) according to the individual's task distribution (i.e., the occurrence and duration of the different tasks within the job; task proportions designate the duration of each task relative to the whole working time) [27]. Task exposures may be aggregated in a task exposure matrix (TEM) in the same way as job exposures may be aggregated in a job exposure matrix [26]. Task-based exposure assessment is particularly likely to be successful if there are large exposure differences between the tasks, and it has previously been shown that there are significant differences in biomechanical exposures between the tasks of male house painters [28].

The aim of this study was to determine to which degree there are sex differences in task distribution and task exposures with respect to postures and movements of the wrist, shoulder, and head among Danish house painters, and to establish sex-specific TEMs. The study was conducted as a part of the SHARM (Shoulder-Hand-ARM) study. The study aims were fulfilled.

## Methods

This study combined questionnaire information on task distribution with task exposures measured by goniometry and inclinometry. All data was collected from Danish house painters who receive professional training for 3 ½ years.

### Questionnaire

In 2011, we sent (by postal mail) a questionnaire about musculoskeletal health and work to all members of the Painters' Union in Denmark, who were born in 1940 or later (N = 9364, 3128 (33.4%) women and 6236 (66.6%) men). A maximum of two reminders were sent. One question concerned the task distribution during a typical week. The question listed the 11 most common tasks according to representatives of the Painters' Union, and a task labelled “other”: 1) removing wallpaper.; 2) levelling; 3) sanding (hand); 4) sanding (giraffe drywall sander); 5) painting (brush); 6) painting (roll); 7) spraying; 8) hanging wallpaper.; 9) covering, carrying materials and equipment, or cleaning; 10) pause; 11) driving; 12) other. The question was phrased: “*This question regards your work after 1990. The question is a bit difficult. Try your best to make the hours add up to your total working hours in a typical week. Please, state the average number of hours you have spent on each task. Start by writing 0 for tasks that you have usually not performed. Then distribute your working hours so that the numbers add up to your total working hours in a typical week*”. The participants were encouraged to draft and add up the hours before filling in the questionnaire. We converted the self-reported task distribution to task proportions for each individual and tested for sex differences in the average percentage of time spent on each task. Due to large sex differences in mean age, the questionnaire data was analysed divided into 5 age-groups.

### Exposure measurements

All painters' workshops (N = 267) in the Capital Region of Denmark were identified in the Danish Central Business Register. In a random order, we contacted the workshops by postal mail, followed by a phone-call, and asked them if 1–4 of their house painters (preferably the same number of men and women) would be willing to participate in exposure measurements during one whole working day per person. This procedure was continued until 25 men and 25 women were included. Originally, we intended to ask the companies to provide a list of all employed house painters, so that we could ask a random sample to participate [28], but in many cases there were only a few employees or few employees expressed willingness to participate, so we gave up this procedure.

After contact to 53 companies, 22 companies had agreed to participate, and the predetermined number of participants had been achieved. For each participant, one whole day measurement was performed on an ordinary working day from Monday to Thursday (Friday was avoided because it normally had fewer working hours). The measurements were performed in the period from May 2011 to March 2012. Only right handed persons without current upper extremity complaints were included. Investigators met with the participants at their worksite or at the workshop. Preparation of the measurements was carried out by one of two investigators and lasted approximately one hour including questions on background characteristics. The time spent on preparation of the measurements was paid for by the employer. After preparation of the measurements, the investigator left and then returned at the end of the day to remove the equipment. During the measurements, the participants filled in the clock time for changes between tasks in a logbook. The tasks were predefined and corresponded to the ones in the questionnaire. Each participant was given a synchronized clock radio so that the time could be read from a digital display. Thus, the measurements could subsequently be divided according to tasks.

During the data collection four tasks had less than 5 individual measurements for both the group of men and the group of women respectively. These tasks were: 1. removing wallpaper; 4.sanding (giraffe); 7. spraying; and 8. hanging wallpaper. Instead these measurements were added to task number 12 containing other tasks than the predefined ones. In order to avoid attenuation of differences between task exposures due to imprecise indication of time for task-change, two minutes were excluded from the measurements at the beginning and end of each task before we calculated task exposures for the TEMs. For the overall job exposure “total work”, the entire recording was used. If a participant performed a given task more than once, the recordings were pooled.

Biaxial goniometers (SG75, Biometrics Ltd, Newport, UK) were used for measuring wrist postures and movements. The goniometers were placed on the dorsal side of each wrist with the distal part over the third metacarpal bone and the proximal part in the midline between ulna and radius [29]–[31]. Initially, recordings were made in the anatomical reference position in order to define the neutral posture, i.e., 0° of flexion/extension and radial/ulnar deviation. This was done with the participants sitting down, resting their lower arms and hands on a table with their palms facing down.

Triaxial inclinometers (Logger Teknologi HB, Åkarp, Sweden) were used for measuring inclination of the head and elevation of the upper arms [32]. The inclinometers were placed on the forehead and on the lateral side of both upper arms just beneath the protrusion of the middle deltoid muscle [33]. To define a neutral posture, reference positions were initially recorded. For the head, the participants were standing, looking at an object 2 to 4 meters away at eye-level. For the arms, the participants were sitting sideways on a chair leaning against the backrest with their arm hanging vertically, holding a 2 kg dumbbell in their hand [33].

The goniometry and inclinometry data was recorded by two person-worn data-loggers (Logger Teknologi HB, Åkarp, Sweden) with a sampling frequency of 20 Hz [34]. Data from both sides were analysed. For each participant, the exposure measures were derived for “total work” and for the different tasks. For the wrist, the 10^th^, 50^th^, and 90^th^ percentiles for the flexion/extension movement were used to represent the extended, median, and flexed wrist-posture, respectively. The corresponding percentiles for radial/ulnar deviation were used to represent radial, median, and ulnar deviation, respectively. The 5^th^–95^th^ interpercentile range described the range of motion. Movement velocity was represented by its median, and the mean power frequency (MPF) was used as a measure of repetitiveness; for a strictly cyclic movement, MPF is identical to the inverse value of the cycle time [29]. Non-neutral postures of the wrist were defined as the % time with angles exceeding 45° flexion/extension or 20° ulnar/radial deviation.

For head posture the 1^st^, 50^th^, and 90^th^ percentiles were used to represent the backward, median, and forward inclination, respectively (49). Upper arm elevation was characterized by the 99^th^ percentile (the angle that is exceeded for 1% of the time) and the % time spent with an elevation above 90°. As measures of variation, the 5^th^–95^th^ interpercentile range was calculated for each minute. The mean value of these one minute samples was defined as the “within-minute variation” and the standard deviation as the “between-minute variation” [35].

### Statistical analyses

We used Cochran-Mantel-Haenszel statistics to test for statistically significant (p<0.05) sex differences in task proportions within each age-group. To evaluate sex differences in task exposures, we used an unpaired double-sided t-test with post-hoc Bonferroni correction. Differences between task exposures for the right and left side were tested using a paired double-sided t-test. Statistical analyses were made using SAS statistical software (v9.2 Cary, NC, USA).

### Ethics Statement

The study was accepted by the Regional Scientific Ethics Committee, Capital region of Denmark (j.no.: H-C-FSP-2010-036). Participants in the exposure measurements gave informed written consent. Permission to store the personal information about the participants was given by the Danish Data Protection Agency (j.no.: 2010-41-5325). Data was anonymised before performing the analyses.

## Results

### Questionnaire

The proportion who responded was 53% (n = 4957), 59% among women and 50% among men. The mean age of the non-responders was 31 years among men and 41 years among women. Table 1 shows characteristics of the questionnaire respondents. The age distribution differed considerably between men and women with a higher percentage of older men.

**Table 1:** Characteristics of questionnaire respondents and participants in exposure measurements.

	Questionnaire respondents						Participants in exposure measurements					
	Men (n = 3124) respondents			Women (n = 1833) respondents			Men (n = 25)			Women (n = 25) measured		
	Mean	SD	%	Mean	SD	%	Mean	SD	%	Mean ()	SD	%
Seniority (years)	27.1	14.3	-	13.8	10.3	-	22.8	14.4	-	8.6	7.2)	-
Working hours per week	36.8	2.8	-	36.4	3.2	-	37.0	0	-	37.0	0	-
**≤30**	-	-	1	-	-	3	-	-	0	-	-	0
**>30 - ≤35**	-	-	1	-	-	6	-	-	0	-	-	0
**>35**	-	-	98	-	-	91	-	-	100	-	-	100
Age (years)	49.7	14.8	-	35.2	11.4	-	45.1	13.1	-	31.9	10.5	-
**17**–**25**	-	-	9	-	-	24	-	-	4	-	-	28
**26**–**35**	-	-	12	-	-	32	-	-	20	-	-	44
**36**–**45**	-	-	17	-	-	25	-	-	28	-	-	16
**46**–**55**	-	-	16	-	-	13	-	-	24	-	-	8
**56**–**70**	-	-	46	-	-	6	-	-	24	-	-	4
Height (cm)	178.7	7.3	-	167.4	6.3	-	180.4	6.0	-	166.2	4.9	-
Weight (kg)	84.2	14.7	-	70.4	14.3	-	85.6	14.2	-	65.8	12.7	-
Body mass index (kg/m^2^)	26.4	4.3	-	25.1	4.9	-	23.7	3.9	-	19.8	3.9	-
Left hand dominance	-	-	9	-	-	10	-	-	0	-	-	0
Right hand dominance	-	-	79	-	-	83	-	-	100	-	-	100
No dominant hand	-	-	12	-	-	8	-	-	0	-	-	0

**Figure 1:**
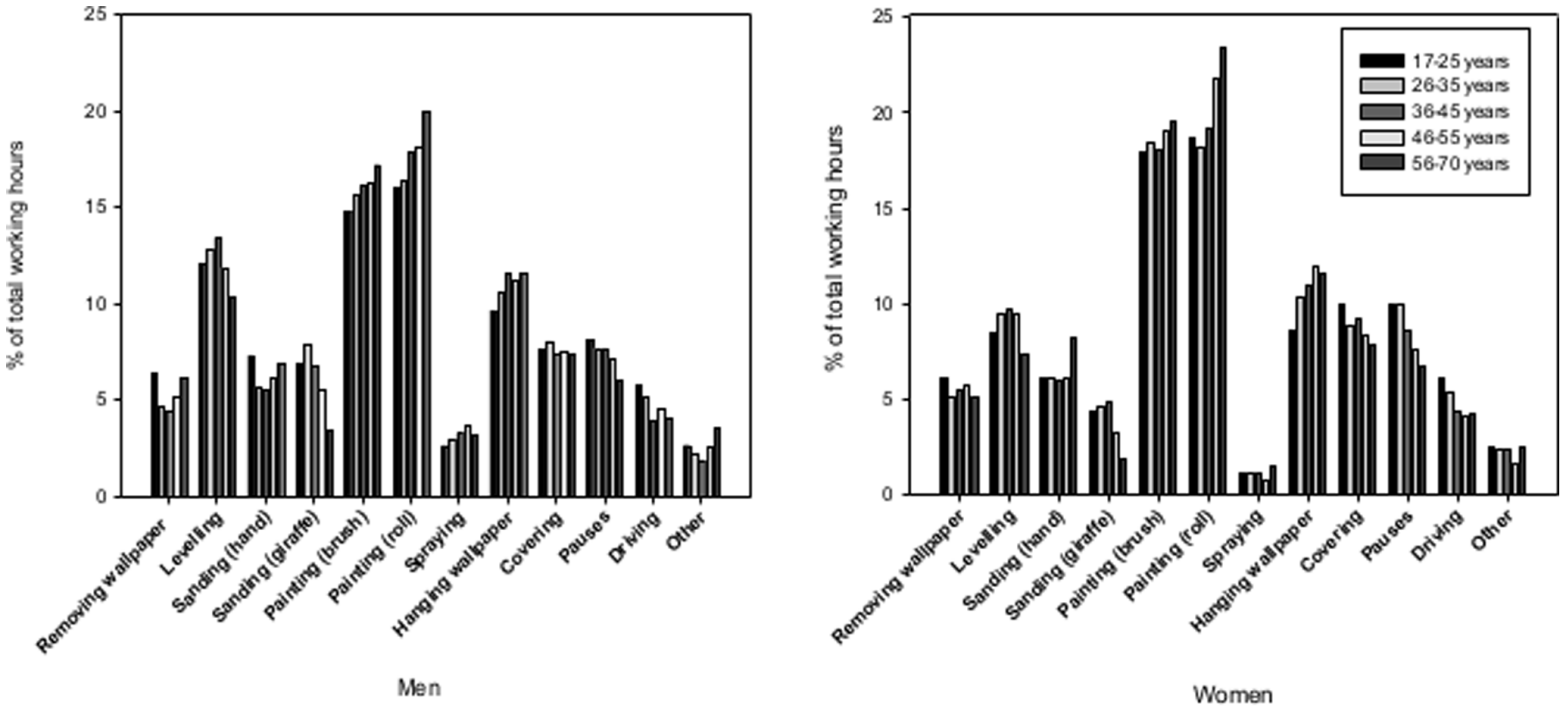
Mean task distribution for men and women, respectively, by age-group.

Figure 1 displays mean task proportions for men and women, respectively, according to age-group. Within tasks, mean task proportions differed by up to 5% between age-groups. Age-group patterns were similar for men and women.

**Figure 2:**
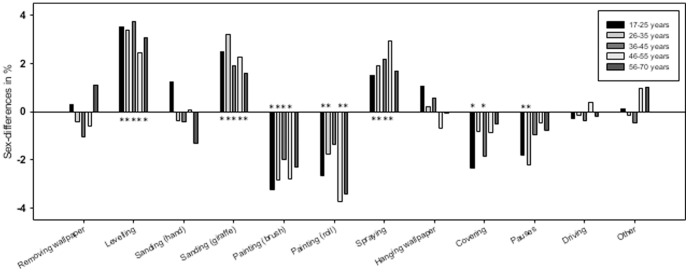
Sex differences in mean task proportions, by age-group. Women are reference-group.* Indicates a statistically significant (p<0.05) sex difference in the specific age-group.

Figure 2 presents sex differences in task proportions, according to age-group. Some statistically significant differences between men and women were found, but none exceeding ±4%. Men had higher task proportions for levelling, sanding (giraffe), and spraying, whereas women had higher task proportions for painting with brush and roll.

### Exposure measurements

Table 2 shows the TEM for postures and movements of the right wrist for each sex. “Total work” represents the overall job exposure. No statistically significant sex differences were observed. For both sexes, there were clear differences in exposures between tasks. For example, the median angular velocity for flexion/extension during painting (brush) was approximately 4°/s less than for sanding (hand) for both men and women. The median angular velocity was approximately 50% higher for flexion/extension of the wrist than for ulnar/radial deviation in all tasks due to the higher range of motion for flexion/extension than for ulnar/radial deviation. MPF was approximately the same for flexion/extension as for deviations since this measure is sensitive to the frequency, but not to the amplitude of the movements. Some measures seem to reflect the same task properties to a great extent. For example the 50^th^ percentiles for flexion/extension and non-neutral postures showed the same difference between men and women within tasks.

**Table 2:** Task exposure matrix for postures and movements of the right wrist for each sex.

			Levelling		Sanding (hand)		Painting (brush)		Painting (roll)		Covering		Driving		Other		Total work		Pause	
			Mean	SD	Mean	SD	Mean	SD	Mean	SD	Mean	SD	Mean	SD	Mean	SD	Mean	SD	Mean	SD
**Flexion/extension**																				
Percentile (°)	10^th^	Men	−57	16	−47	6	−50	12	−43	11	−42	14	−46	14	−45	18	−48	12	−44	17
		Women	−46	8	−51	9	−52	10	−47	9	−52	12	−47	13	−48	8	−50	8	−48	8
	50^th^	Men	−26	10	−15	3	−23	10	−17	11	−17	18	−20	13	−20	14	−20	11	−15	12
		Women	−18	8	−26	13	−25	7	−23	8	−23	13	−13	9	−21	11	−22	8	−22	10
	90^th^	Men	0	10	10	2	3	9	8	13	10	15	5	13	5	12	7	11	12	15
		Women	6	8	0	11	5	9	6	11	5	9	11	8	7	10	7	8	9	14
Range of motion	5^th^–95^th^	Men	75	10	74	9	69	10	65	12	66	20	67	12	65	11	72	7	70	17
		Women	67	4	65	11	74	14	70	15	72	13	76	14	74	9	75	10	74	11
Median velocity (°/s)		Men	18.1	8	19.3	5	15.5	7	17.6	6	13.3	8	10.0	5	14.0	8	14.5	5	5.5	4
		Women	21.2	7	19.1	4	15.3	5	16.3	5	14.2	8	7.9	3	14.5	6	14.6	4	4.8	4
Repetitiveness (MPF; Hz)		Men	.28	.05	.29	.04	.26	.06	.30	.08	.29	.08	.28	.06	.29	.03	.27	.04	.29	.06
		Women	.33	.05	.28	.06	.25	.04	.28	.06	.26	.06	.24	.05	.28	.06	.27	.04	.20	.03
**Ulnar/radial deviation**																				
Percentile (°)	10^th^	Men	−8	5	−19	5	−15	10	−18	9	−9	8	−9	6	−13	11	−15	9	−15	9
		Women	−13	8	−21	16	−21	11	−22	9	−20	12	−17	9	−20	11	−19	11	−18	10
	50^th^	Men	9	3	−4	5	1	11	−2	7	6	9	6	6	4	10	1	9	0	9
		Women	3	6	−4	16	−5	10	−5	9	−4	8	−2	8	−4	11	−2	10	−3	10
	90^th^	Men	27	5	11	4	18	12	17	10	19	10	21	7	19	9	18	9	14	9
		Women	19	6	14	12	12	10	12	8	13	8	13	7	11	10	14	9	12	10
Range of motion	5^th^–95^th^	Men	44	11	40	3	42	8	44	9	36	10	38	9	42	7	42	8	37	8
		Women	41	8	44	7	44	5	43	6	42	9	38	8	41	6	43	6	39	7
Median velocity (°/s)		Men	10.9	6	11.5	3	10.8	6	12.9	7	8.1	4	5.7	3	8.8	4	9.0	3	3.6	3
		Women	12.3	5	15.3	6	9.9	3	13.1	6	8.9	7	4.8	1	8.9	4	9.2	3	3.2	2
Repetitiveness (MPF; Hz)		Men	.29	.06	.33	.04	.28	.06	.30	.08	.31	.06	.29	.08	.27	.03	.28	.05	.21	.06
		Women	.33	.06	.30	.06	.27	.06	.31	.07	.28	.07	.25	.06	.28	.06	.28	.06	.20	.05
**Combined wrist postures**																				
Non-neutral postures (% time)		Men	43	16	20	2	33	19	28	14	24	22	29	18	31	18	30	15	22	14
		Women	26	9	39	21	36	13	32	13	34	16	25	9	28	17	32	12	31	15
**Number of recordings**		Men	5	-	5	-	14	-	13	-	12	-	8	-	10	-	24	-	23	-
		Women	7	-	8	-	17	-	15	-	16	-	8	-	15	-	25	-	25	-
**Mean recording duration** (minutes)		Men	82	-	76	-	162	-	138	-	46	-	45	-	94	-	280	-	48	-
		Women	127	-	51	-	149	-	102	-	55	-	41	-	78	-	309	-	59	-

Data are displayed for the 7 tasks that constitute the work. Additionally, data are shown for total work and pause. For flexion/extension and ulnar/radial deviation, positive angles denote flexion and ulnar deviation, respectively, and negative angles extension and radial-deviation, respectively, [SD = standard deviation; MPF =  mean power frequency].

Table 3 shows the TEM for postures and movements of the head and right upper arm for each sex; again, job exposures are presented as well. There were no statistically significant sex differences. Between-minute variation was higher for “total work” than for any of the tasks that constituted the work. This shows that, unlike the rest of the exposure measures, job exposures in terms of between-minute variation cannot even approximately be derived by a straight forward time weighting of task exposures.

**Table 3:** Task exposure matrix for postures and movements of the head and right upper arm for each sex.

			Levelling		Sanding (hand)		Painting (brush)		Painting (roll)		Covering		Driving		Other		Total work		Pause	
			Mean	SD	Mean	SD	Mean	SD	Mean	SD	Mean	SD	Mean	SD	Mean	SD	Mean	SD	Mean	SD
**Head inclination**																				
Percentile (°)	1^st^	Men	−46	12	−39	14	−45	13	−53	12	−26	22	−16	10	−38	13	−45	14	−22	14
		Women	−40	6	−40	17	−45	14	−53	15	−23	15	−18	4	−40	12	−47	12	−21	14
	50^th^	Men	18	14	24	8	16	16	8	13	25	16	15	14	19	15	17	14	16	13
		Women	16	5	18	11	15	11	7	22	27	12	13	9	17	11	17	8	13	12
	90^th^	Men	64	11	54	6	54	15	50	12	52	17	44	14	51	14	52	13	43	15
		Women	55	8	55	11	53	14	54	12	54	12	40	11	51	11	53	10	36	14
**Right upper arm elevation**																				
99^th^ percentile (°)		Men	136	11	123	12	123	12	121	23	89	27	90	17	121	25	127	13	90	27
		Women	131	14	124	30	127	18	126	21	95	22	94	23	126	15	128	15	84	23
>90° (% time)		Men	15	8	6	2	12	9	8	7	2	2	4	8	6	5	9	4	2	3
		Women	9	4	11	6	12	8	11	7	3	4	3	6	10	11	9	5	1	1
Within-minute variation (°)^a^		Men	75	18	62	2	68	15	65	17	42	10	39	11	59	20	62	10	32	11
		Women	73	16	75	21	70	20	68	16	47	11	46	16	62	13	62	11	30	15
Between-minute variation (°)^a^		Men	29	5	29	6	28	6	26	9	18	8	18	6	28	6	31	5	20	7
		Women	31	5	26	12	29	7	27	7	19	8	19	3	30	5	32	6	22	7
Median velocity (°/s)		Men	51.6	16	59.9	13	47.0	17	52.5	19	47.0	24	33.6	12	38.5	18	42.9	12	17.0	19
		Women	59.9	20	66.4	25	43.2	14	50.8	13	48.4	25	29.4	11	43.9	18	43.7	13	10.1	9
**Number of recordings**		Men	5	-	6	-	17	-	14	-	12	-	9	-	10	-	25	-	24	-
		Women	7	-	8	-	17	-	15	-	16	-	8	-	15	-	25	-	25	-
**Mean recording duration** (minutes)		Men	88	-	95	-	158	-	130	-	52	-	51	-	124	-	313	-	52	-
		Women	158	-	51	-	149	-	102	-	55	-	41	-	78	-	318	-	61	-

Data are displayed for the 7 tasks that constitute the work. Additionally, data are shown for total work and pause. For flexion/extension, positive angles denote flexion and negative angles extension. [SD = standard deviation].

a The measures of variation were calculated from the 5^th^–95^th^ interpercentile range for each minute.

For both sexes, median velocity was distinctively lower for the wrist and upper arms in the tasks “driving” and “pause” than in any other task (tables 2 and 3).

Job exposures differed statistically significantly (p<0.05) between left and right sides. For flexion/extension and ulnar/radial deviation, both men and women had a higher median velocity and MPF on the right side; the same was present for median velocity of shoulder elevation. Sex-specific TEMs were also established for the left side, i.e., the non-dominant side since all participants were right-handed. The TEMs for the left side are available from the supporting information file (Table S1).

## Discussion

This study showed only minor sex differences in task distribution and task-specific postures and movements among Danish house painters. There was a considerable difference in age and seniority between male and female respondents. Thus, reported task distributions for men could reflect a longer period back in time, where task distributions may have differed from nowadays. However, we controlled for effects of age by stratification, and the age-dependent patterns within each task were quite similar for men and women. The proportion who returned the questionnaire was relatively low, and responders and non-responders differed from each other with respect to sex and age distribution, but we think that the age-specific comparisons counteracted the risk of non-response bias with respect to assessing sex differences in task distributions.

There is a potential for recall bias with a tendency for overestimating time proportions spent on highly exposed tasks; in particular, women might tend to overestimate time proportions spent on force demanding tasks because they use more force relative to their maximum than men do [23], [24]. Even if this was the case, the slight differences in task proportions, which we identified, seemed to be in a direction of men doing more of the tasks that require high force [23].

During the whole day exposure measurements, a certain task could be performed by few persons and the total time spent on the task could be very short. Based on recent recommendations [35], we decided on a minimum of 5 measurements for each sex per task. In case of an insufficient number of measurements for a specific task, the task was added to the task “other”, which diminished the number of tasks from 12 to 8. An alternative procedure could have been to supplement the whole day measurements by task-specific measurements until the pre-specified number of measurements per task was met, but this would have been time consuming beyond our resources. As a tentative rule of thumb, a minimum of 120 minutes of measurement per task has been recommended when constructing a TEM [36]. For all 8 tasks in our TEM, the total duration of task exposure measurements was well above this limit.

Self-reported logbook data on task occurrence and duration may be less precise than direct observation [37]. In an attempt to exclude potential overflow between tasks due to imprecise timing, we decided to exclude two minutes in the beginning and end of each task measurement. This step caused only minor changes, which indicates a precise overall reporting. We did not apply a minimum duration of task recording per period, but due to the 2 minutes cut-off, recordings lasting less than 4 minutes were discarded.

Regarding non-neutral wrist postures, our limit for radial deviation (20°) was set higher than reported normative data on range of motion [38]. This was done because flexion/extension and ulnar/radial deviation movements are coupled [39], [40] so that during normal activity, radial deviation angles exceed the range of motion of radial deviation in a constrained neutral flexion/extension angle. This is caused by an oblique orientation of the mechanical axes in relation to the anatomical axes of the wrist [41]. Our choice of ±45° for flexion/extension is consistent with the symmetrical properties of these movements and well within reported range of motions [38]. However, the contributions of flexion/extension to non-neutral postures were almost entirely due to excessive extension. This corresponds well with the 50^th^ percentile for flexion/extension which showed that all tasks were performed with a generally extended wrist; in this posture the fingers can exert more power in the grip. Extended wrist postures have been shown to produce particularly high carpal tunnel pressures, which has been linked to the development of carpal tunnel syndrome and tendon related disorders [42]–[44]. Especially, the combination of non-neutral postures and a high use of force has been reported as a risk factor for developing MSDs in the upper extremity [45]. Contrary to this, a recent study showed a protective effect of wrist extension >43° during heavy grip, but this study mainly concerned de Quervain's disease and the study had very few cases [46].

Exposure measures for work with elevated arms are commonly derived from the right upper tail of the amplitude distribution function (ADF) of the elevation angle. A high percentile, e.g. the 99^th^, can be selected, and the corresponding angle, xx°, derived from the ADF. The interpretation of this measure is that for 1% of the time, the elevation angle exceeded xx°. Alternatively, a high elevation threshold, e.g. 90° can be chosen, and the % time above this angle, yy%, can be derived; the interpretation is that for yy% of the time, the elevation exceeded 90°. Percentiles are commonly used to express neutral and extreme postures without any assumptions about the pathophysiological mechanisms [47]–[52]. A disadvantage with percentiles is that time weighting of task exposure is an approximation [47], which may lead to bias for short measurements [36]. Thresholds are more intuitive and are generally considered to be easily assessable from low-cost observations and thus preferable for epidemiological studies and work place visits e.g. by occupational physicians. However, observations may be as resource demanding as measurements, and may introduce bias [53]. The choice of thresholds may be based on hypotheses on pathogenesis [27] and safe lower levels of exposure. Exposures below the threshold are disregarded. For upper arm elevation >90°, the SD in our material exceeded the group mean for some of the tasks, e.g. driving (table 3). This is a drawback because it shows that the confidence intervals for these task exposures are wide.

Upper extremities MSDs have been demonstrated to be more prevalent in the dominant arm [13], [54]. This distinction is also evident within the house painters' trade [25]. Our tables presented exposure measurement data from the right side, which was the dominant side for all participants, by design. We think that these task exposures can be assigned to the dominant side for left-handed persons because house painters are not constrained to use the right hand due to the design of tools or characteristics of worksites.

Small, but statistically significant sex differences were found for some tasks when comparing the self-reported task distributions. Several studies have addressed the problem of sex-segregated tasks in relation to sex differences in MSDs within a job [9], [24], [55]–[59]. In our study, task-specific postures and movements did not show statistically significant sex differences, and it could therefore be considered to use a common TEM for men and women, even in studies of sex differences in MSDs. However, the sex-specific TEMs enable inclusion of the derived sex differences, when estimating the individual exposure in future epidemiological studies. Moreover, the TEMs can be extended with sex-specific data regarding use of force (23).

In conclusion, sex-specific TEMs were constructed for the head and the dominant and non-dominant wrist and upper arm. Only minor sex differences were found in self-reported task distributions and objectively measured task-specific postures and movements of the upper extremities. Thus, the house painters' trade seems well suited to investigate sex differences in vulnerability to exposures that may cause upper extremity MSDs.

## Supporting Information

Table S1 **Task exposure matrix for postures and movements of the left wrist for each sex.** Data are displayed for the 7 tasks that constitute the work. Additionally, data are shown for total work and pause. For flexion/extension and ulnar/radial deviation, positive angles denote flexion and ulnar deviation, respectively, and negative angles extension and radial-deviation, respectively, [MPF =  mean power frequency].
(DOCX)
