# Supplementary material for: Sex Differences in Task Distribution and Task Exposures among Danish House Painters: An Observational Study Combining Questionnaire Data with Biomechanical Measurements
Source: PLoS One. 2014 Nov 3;9(11):e110899. doi: 10.1371/journal.pone.0110899 (PMC4218834; doi:10.1371/journal.pone.0110899)
Supplement: Table S1 — Task exposure matrix for postures and movements of the left wrist for each sex. Data are displayed for the 7 tasks that constitute the work. Additionally, data are shown for total work and pause. For flexion/extension and ulnar/radial deviation, positive angles denote flexion and ulnar deviation, respectively, and negative angles extension and radial-deviation, respectively, [MPF = mean power frequency]. (DOCX) [file pone.0110899.s001.docx]

| Table S1 | | | | | | | | | | | | | | |
| --- | --- | --- | --- | --- | --- | --- | --- | --- | --- | --- | --- | --- | --- | --- |
|  | | |  | Full leveling | Sanding (by hand) | Painting (brush) | Painting (roll) | Covering, carrying and cleaning | | Driving | | Other | Total work | Pause |
| **Flexion/extension** | | |  |  |  |  |  |  | |  | |  |  |  |
|  | Percentile (°) | 10^th^ | Men | -47 (8) | -54 (10) | -50 (7) | -50 (14) | -42 (12) | | -48 (10) | | -50 (10) | -49 (8) | -45 (15) |
|  |  |  | Women | -52 (15) | -60 (17) | -53 (13) | -52 (11) | -47 (13) | | -48 (11) | | -48 (9) | -51 (10) | -46 (8) |
|  |  |  |  |  |  |  |  |  | |  | |  |  |  |
|  |  | 50^th^ | Men | -20 (7) | -21 (7) | -20 (8) | -17 (9) | -14 (8) | | -22 (12) | | -21 (9) | -18 (8) | -15 (10) |
|  |  |  | Women | -21 (11) | -27 (14) | -23 (10) | -25 (11) | -19 (9) | | -18 (10) | | -21 (11) | -21 (10) | -18 (9) |
|  |  |  |  |  |  |  |  |  | |  | |  |  |  |
|  |  | 90^th^ | Men | 2 (9) | 2 (11) | 3 (12) | 5 (11) | 9 (9) | | 10 (13) | | 5 (12) | 6 (11) | 13 (16) |
|  |  |  | Women | 6 (9) | 0 (14) | 6 (13) | 3 (14) | 7 (9) | | 6 (9) | | 7 (11) | 8 (11) | 12 (14) |
|  |  |  |  |  |  |  |  |  | |  | |  |  |  |
|  |  | 95-5^th^ | Men | 64 (7) | 76 (11) | 70 (10) | 70 (11) | 74 (16) | | 73 (12) | | 70 (12) | 74 (6) | 73 (18) |
|  |  |  | Women | 77 (9) | 76 (19) | 77 (15) | 72 (15) | 70 (16) | | 72 (16) | | 75 (12) | 77 (11) | 74 (13) |
|  |  | |  |  |  |  |  |  | |  | |  |  |  |
|  | Median velocity (°/s) | | Men | 11 (4) | 10 (3) | 10 (4) | 12 (8) | 10 (3) | | 9 (4) | | 9 (4) | 9 (4) | 6 (5) |
|  |  |  | Women | 11 (4) | 15 (4) | 8 (5) | 12 (6) | 12 (5) | | 8 (3) | | 10 (3) | 10 (4) | 4 (2) |
|  |  | |  |  |  |  |  |  | |  | |  |  |  |
|  | Repetitiveness (MPF; Hz) | | Men | .26 (.03) | .23 (.05) | .20 (.04) | .24 (.08) | .23 (.05) | | .25 (.05) | | .22 (.03) | .22 (.04) | .19 (.05) |
|  |  |  | Women | .22 (.03) | .24 (.06) | .20 (.05) | .24 (.06) | .27 (.07) | | .26 (.07) | | .24 (.03) | .22 (.04) | .18 (.04) |
| **Ulnar/radial deviation** | | |  |  |  |  |  |  | |  | |  |  |  |
|  | Percentile (°) | 10^th^ | Men | -12 (9) | -22 (5) | -21 (10) | -22 (9) | -16 (7) | | -16 (7) | | -17 (9) | -21 (9) | -21 (11) |
|  |  |  | Women | -16 (7) | -21 (10) | -28 (11) | -22 (10) | -20 (11) | | -19 (8) | | -23 (9) | -22 (9) | -21 (9) |
|  |  |  |  |  |  |  |  |  | |  | |  |  |  |
|  |  | 50^th^ | Men | 0 (7) | -7 (5) | -5 (8) | -7 (8) | -4 (7) | | -4 (8) | | 3 (8) | -5 (8) | -5 (9) |
|  |  |  | Women | -2 (5) | -5 (9) | -7 (8) | -5 (8) | -3 (6) | | -5 (7) | | -4 (9) | -4 (7) | -5 (9) |
|  |  |  |  |  |  |  |  |  |  | |  | |  |  |
|  |  | 90^th^ | Men | 18 (5) | 6 (5) | 12 (10) | 9 (10) | 11 (8) | 10 (9) | | 12 (8) | | 11 (9) | 9 (9) |
|  |  |  | Women | 13 (4) | 9 (9) | 9 (8) | 10 (8) | 11 (6) | 8 (6) | | 12 (7) | | 11 (7) | 10 (9) |
|  |  |  |  |  |  |  |  |  |  | |  | |  |  |
|  |  | 95-5^th^ | Men | 38 (6) | 37 (3) | 43 (12) | 39 (7) | 36 (9) | 32 (4) | | 36 (5) | | 42 (9) | 39 (10) |
|  |  |  | Women | 39 (5) | 42 (8) | 48 (11) | 42 (6) | 42 (13) | 35 (5) | | 43 (5) | | 43 (6) | 39 (9) |
|  |  | |  |  |  |  |  |  |  | |  | |  |  |
|  | Median velocity (°/s) | | Men | 7 (2) | 6 (1) | 5 (3) | 7 (4) | 6 (2) | 5 (2) | | 5 (2) | | 5 (2) | 3 (3) |
|  |  |  | Women | 6 (2) | 10 (4) | 5 (4) | 8 (4) | 7 (3) | 5 (2) | | 6 (2) | | 6 (3) | 2 (2) |
|  |  | |  |  |  |  |  |  | |  | |  |  |  |
|  | Repetitiveness (MPF; Hz) | | Men | .24 (.02) | .23 (.03) | .21 (.05) | .25 (.07) | .23 (.05) | | .28 (.07) | | .23 (.04) | .23 (.05) | .20 (.06) |
|  |  |  | Women | .24 (.05) | .27 (.06) | .19 (.05) | .25 (.06) | .25 (.07) | | .27 (.05) | | .23 (.04) | .23 (.05) | .19 (.03) |
|  | | |  |  |  |  |  |  | |  | |  |  |  |
| Number of recordings | | | Men | 5 | 5 | 14 | 13 | 12 | | 8 | | 10 | 25 | 23 |
|  |  |  | Women | 7 | 8 | 17 | 15 | 16 | | 8 | | 15 | 25 | 25 |
|  | | |  |  |  |  |  |  | |  | |  |  |  |
| Mean recording duration in minutes | | | Men | 88 | 102 | 141 | 128 | 49 | | 55 | | 118 | 280 | 45 |
|  |  |  | Women | 158 | 51 | 149 | 102 | 55 | | 41 | | 77 | 317 | 61 |
| **Values are presented in means (SD)** | | | | | | | | | | | | | | |
